# Evaluating support for the debrief process for paediatric oncology trainee doctors and newly appointed consultants after child death

**DOI:** 10.1136/bmjpo-2026-004488

**Published:** 2026-06-24

**Authors:** Upeka Senanayake, Phoebe Aubugeau-Williams, Gemma Barnard

**Affiliations:** 1 Paediatric Haematology/Oncology/BMT, Cambridge University Hospitals NHS Foundation Trust, Cambridge, UK; 2 Department of Paediatrics, University of Cambridge, Cambridge, UK

**Keywords:** Psychology

 Careers in paediatric haematology and oncology are both rewarding and emotionally challenging. Managing the death of young patients is one of the most significant stressors, contributing to high burnout rates. Burnout is recognised as an occupational illness, and affected individuals may be compelled to leave their roles.^1^ The 2024 General Medical Council (GMC) survey of over 47 500 UK medical trainees, of which 61% completed burnout-related questions, reported over half (52%) felt emotionally exhausted to a high or very high degree,^2^ highlighting an urgent need for improved well-being support.

The GMC’s *Good Medical Practice* advises doctors to monitor their own health and well-being, and seeking help when needed,^3^ though wider factors are required to improve staff retention and patient safety, such as prioritising workload management, improving working conditions and fostering inclusive, supportive environments. Recognising the impact of an ethically-challenging case involving child death on trainee well-being, a principal treatment centre (PTC) introduced confidential debrief sessions led by a pastoral general practitioner to provide emotional support.

The study aimed to assess the psychological support needs of trainees and early-career consultants by conducting a two-phase local and national survey. A local survey of paediatric oncology trainee doctors at a PTC was followed by a national survey, distributed via the Children’s Cancer and Leukaemia Group Connect and the Paediatric Oncology Trainee Group. This work was conducted as a service evaluation study; therefore, formal ethical approval was not required.

**Figure 1:**
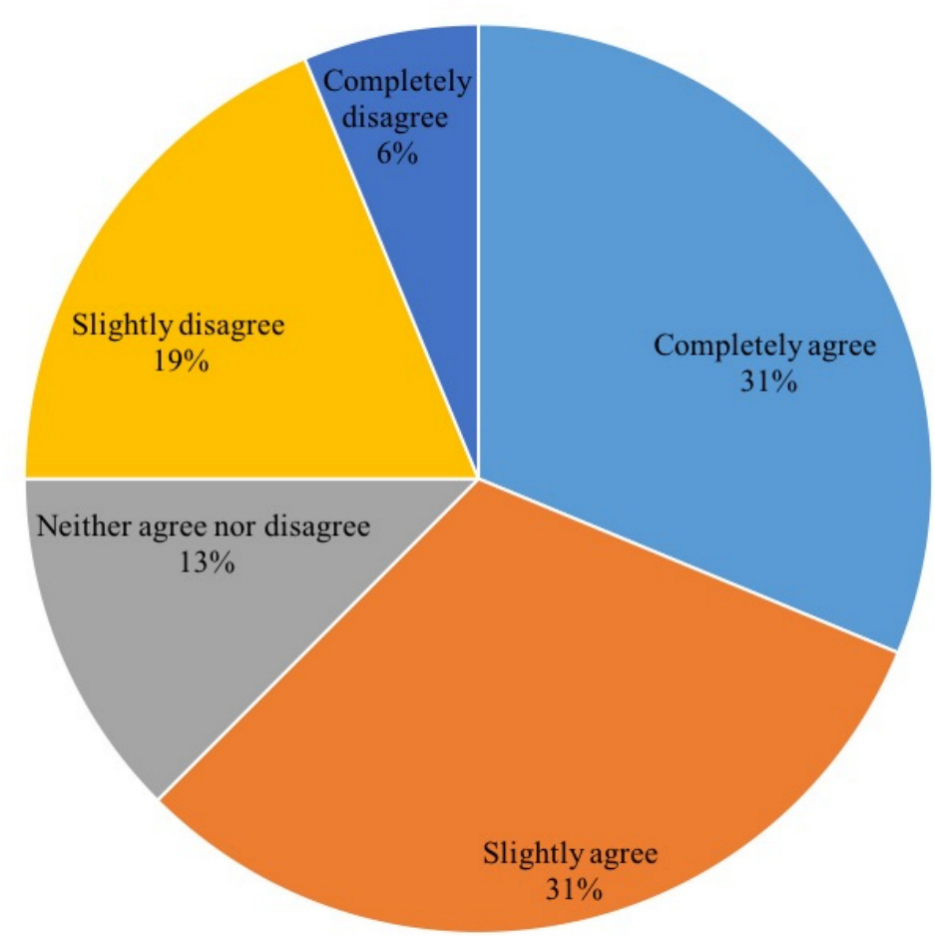
The emotional nature of this rotation had a significant impact on my wellbeing and resilience.

16 doctors responded to the local survey, a range of specialty trainee (ST) 1–3 and ST 4–8 trainees and specialty doctors. Placement dates ranged from 2023 to 2024, besides one outlier in 2020. There was strong recognition of the emotional impact of caring for children with cancer, with all in agreement that the role is emotionally challenging and this had a variable impact on well-being and resilience (figure 1). Temporal analysis and free-text answers revealed the perceived value of regular well-being sessions offered by the trust.

15 resident doctors and early career consultants working in 10 different tertiary paediatric oncology centres completed the national survey, with placement dates ranging from 2020 to 2024, besides one outlier in 2017. All respondents agreed or strongly agreed that caring for children with cancer is emotionally challenging. Witnessing or experiencing patient death, children’s and/or parents’ distress were ranked as having the greatest emotional impact. A majority indicated the emotional nature of this job negatively affects their well-being (53%) and contributes to feelings of burnout (67%). Many felt their emotional needs were unmet and discussed wider cultural factors hindering optimal trainee well-being.

Local and national survey results both reflected a collective acknowledgement of the emotional toll of working in paediatric oncology, as well as the importance of well-being support to optimise doctors’ resilience and allow them to thrive in this environment. Though findings highlighted a need for emotional support at work, including debriefing and senior support, access and availability of this was highly variable nationally. Barriers to seeking and accessing support were identified, including workload pressures and staff shortages, and free-text answers emphasised the benefit of a tailored approach to meet individual needs. Respondents to the local survey valued regular well-being sessions provided by the trust and emotional support through supervision, providing a case for wider implementation.

Previous evidence suggests that emotional support and debriefing sessions improve staff retention among paediatric haematology and oncology doctors.^4^

This study suggests there is a critical need for more structured, accessible and tailored emotional support for UK doctors working in paediatric oncology. Our future aim is to develop a national hub to support the well-being of this group.
