# Supplementary material for: Evaluating support for the debrief process for paediatric oncology trainee doctors and newly appointed consultants after child death
Source: BMJ Paediatr Open. 2026 Jun 24;10(1):e004488. doi: 10.1136/bmjpo-2026-004488 (PMC13295921; doi:10.1136/bmjpo-2026-004488)
Supplement: Supplementary file 1 [file bmjpo-2026-004488.reviewer_comments.pdf]

PEER REVIEW HISTORY

BMJ Paediatrics Open publishes all reviews undertaken for accepted manuscripts. Reviewers are asked to complete a checklist review form and are provided with free text boxes to elaborate on their assessment. These free text comments are reproduced below.

ARTICLE DETAILS

**TITLE (PROVISIONAL)**	EVALUATING SUPPORT FOR THE DEBRIEF PROCESS FOR PAEDIATRIC ONCOLOGY TRAINEE DOCTORS AND NEWLY APPOINTED CONSULTANTS AFTER CHILD DEATH
**AUTHORS**	Senanayake, Upeka<br>Aubugeau-Williams, Phoebe<br>Barnard, Gemma

VERSION 1 – REVIEW

**REVIEWER NAME**	Friedel, Marie
**REVIEWER AFFILIATION**	University of Luxembourg, Faculty of Science, Technology and Medicine
**REVIEWER CONFLICT OF INTEREST**	No Conflict of Interest
**AI DISCLOSURE**	No, I have not used AI
**DATE REVIEW RETURNED**	13-Feb-2026

**GENERAL COMMENTS**	Dear authors. Many thanks for giving me the opportunity to review this research letter on an often under researched topic, namely the emotional support provided to GP's. My only suggestion would to write the first time of appearance the acronyms used (eg. GP, CCLG, ST1), to ensure comprehension for all readers.

**REVIEWER NAME**	KARAGEORGOS, SPYRIDON
**REVIEWER AFFILIATION**	Aghia Sophia Children's Hospital , Pediatric Emergency Department
**REVIEWER CONFLICT OF INTEREST**	No Conflict of Interest
**AI DISCLOSURE**	No, I have not used AI
**DATE REVIEW RETURNED**	02-Apr-2026

**GENERAL COMMENTS**	Interesting study with important implications for trainees and young consultants in how they handle these emotionally impactful cases.<br>Introduction - it would be nice to introduce some data on prior studies in the field especially in paediatric hematology/oncology. What has been already done?<br>Methods<br>Ethics approval or excemption should be provided by authors<br>Results - visualization of results would be nice (e.g., color themed likert scales as supplementary material to assist reader).<br>Discussion - next steps and how authors are planning to use these data to inform Insitutional changes would be valuable for the reader

VERSION 1 – AUTHOR RESPONSE

Dear Reviewers,

Thank you for reviewing our manuscript titled “EVALUATING SUPPORT FOR THE DEBRIEF PROCESS FOR PAEDIATRIC ONCOLOGY TRAINEE DOCTORS AND NEWLY APPOINTED CONSULTANTS AFTER CHILD DEATH ” We are grateful for your insightful comments and have addressed them as follows:

Reviewer 1:

Comment:
Thank you for the opportunity to review this research letter on an often under-researched topic, namely the emotional support provided to GPs. My only suggestion is to define all acronyms (e.g., GP, CCLG, ST1) at first use to ensure clarity for all readers.

Response:
We thank the reviewer for this helpful suggestion. All acronyms are now defined at their first appearance in the manuscript.

Reviewer 2:

Comment:
This is an interesting study with important implications for trainees and young consultants managing emotionally impactful cases. In the Introduction, it would be helpful to include prior studies in the field, particularly in paediatric hematology/oncology. What has already been done?

Response:
We appreciate this suggestion and have now included a relevant prior study as Reference 4 in the discussion section.

Comment:
Ethics approval or exemption should be provided.

Response:
We have now included a statement regarding ethics exemption in the Methods section.

Comment:
Visualization of results would be helpful (e.g., color-themed Likert scales as supplementary material).

Response:
We agree and have added Figure 1 (a pie chart) to improve the visualisation of the results.

Comment:
In the Discussion, outlining next steps and how the findings will inform institutional changes would be valuable.

Response:
Thank you for this suggestion. We have added a sentence addressing future directions; however, to comply with word count limitations, one sentence from the previous version has been removed.

VERSION 2 – REVIEW

**REVIEWER NAME**	Friedel, Marie

**REVIEWER AFFILIATION**	University of Luxembourg, Faculty of Science, Technology and Medicine
**REVIEWER CONFLICT OF INTEREST**	No Conflict of Interest
**AI DISCLOSURE**	No, I have not used AI
**DATE REVIEW RETURNED**	03-May-2026

**GENERAL COMMENTS**	Dear Authors,<br><br>all request by the 2 reviewers have been addressed in the revised version.

**REVIEWER NAME**	KARAGEORGOS, SPYRIDON
**REVIEWER AFFILIATION**	Aghia Sophia Children's Hospital, Pediatric Emergency Department
**REVIEWER CONFLICT OF INTEREST**	No Conflict of Interest
**AI DISCLOSURE**	No, I have not used AI
**DATE REVIEW RETURNED**	22-Apr-2026

**GENERAL COMMENTS**	Authors have responded to suggestions and revised manuscript accordingly.
